# DNMT3A/*miR-129-2-5p*/Rac1 Is an Effector Pathway for *SNHG1* to Drive Stem-Cell-like and Invasive Behaviors of Advanced Bladder Cancer Cells

**DOI:** 10.3390/cancers14174159

**Published:** 2022-08-27

**Authors:** Jiheng Xu, Rui Yang, Jingxia Li, Lidong Wang, Mitchell Cohen, Diane M. Simeone, Max Costa, Xue-Ru Wu

**Affiliations:** 1 Department of Medicine, New York University School of Medicine, New York, NY 10016, USA; 2 Department of Surgery, New York University School of Medicine, New York, NY 10016, USA; 3 Department of Pathology, New York University School of Medicine, New York, NY 10016, USA; 4 Department of Urology, New York University School of Medicine, New York, NY 10016, USA; 5 Veterans Affairs New York Harbor Healthcare System, Manhattan Campus, New York, NY 10010, USA

**Keywords:** urothelial carcinoma, muscle-invasive, stemness, *SNHG1*, Rac1, DNMT3A

## Abstract

**Simple Summary:**

Bladder cancer ranks fourth among the most prevalent cancers in men and is the most expensive cancer to treat on a per-patient basis. The muscle-invasive form of bladder cancer is one of the deadliest cancers, with a 5-year survival rate of only 6% in patients with distant metastasis. Effective therapeutic options remain few and far between. *SNHG1* is a long non-coding RNA that is over-expressed in 95% of muscle-invasive bladder cancers. However, very little information is available about the role and mechanisms of *SNHG1* in bladder tumor formation and progression. Here, we provide experimental evidence establishing that *SNHG1* drives bladder cancer cell invasion and stem-cell-like behaviors through a specific signaling pathway. Our results reveal novel biomarkers predictive of the progression of muscle-invasive bladder cancer and potential new targets for therapeutic intervention.

**Abstract:**

The stem-cell-like behavior of cancer cells plays a central role in tumor heterogeneity and invasion and correlates closely with drug resistance and unfavorable clinical outcomes. However, the molecular underpinnings of cancer cell stemness remain incompletely defined. Here, we show that *SNHG1*, a long non-coding RNA that is over-expressed in ~95% of human muscle-invasive bladder cancers (MIBCs), induces stem-cell-like sphere formation and the invasion of cultured bladder cancer cells by upregulating Rho GTPase, Rac1. We further show that *SNHG1* binds to DNA methylation transferase 3A protein (DNMT3A), and tethers DNMT3A to the promoter of *miR-129-2*, thus hyper-methylating and repressing *miR-129-2-5p* transcription. The reduced binding of *miR-129-2* to the 3′-UTR of *Rac1* mRNA leads to the stabilization of *Rac1* mRNA and increased levels of Rac1 protein, which then stimulates MIBC cell sphere formation and invasion. Analysis of the Human Protein Atlas shows that a high expression of Rac1 is strongly associated with poor survival in patients with MIBC. Our data strongly suggest that the *SNHG1*/DNMT3A/*miR-129-2-5p*/Rac1 effector pathway drives stem-cell-like and invasive behaviors in MIBC, a deadly form of bladder cancer. Targeting this pathway, alone or in combination with platinum-based therapy, may reduce chemoresistance and improve longer-term outcomes in MIBC patients.

## 1. Introduction

The muscle-invasive form of bladder cancer accounts for ~30% of bladder cancers, but it causes almost all bladder cancer deaths [1,2,3,4]. Despite radical cystectomy coupled with pre- and/or post-operative chemotherapy, up to 50% of muscle-invasive bladder cancers (MIBCs) eventually progress to regional and distant metastasis, with 5-year survival rates of 38% and 6%, respectively [1,5]. Although immune checkpoint blockade therapies have shown significant promise in the treatment of metastatic bladder cancer, this approach benefits only ~25% of patients [6]. Multi-omics studies have profiled thousands of human MIBC specimens but have not delineated the driver events for bladder cancer metastasis [7,8,9]. This argues against the existence of a major barrier to the progression from bladder cancer invasion to metastasis. Thus, controlling bladder cancer at the invasive stage remains the key to reducing the number of deaths caused by this disease.

Mounting evidence suggests that stem-cell-like behavior (or stemness) plays a central role in driving cancer cell invasion [10,11]. While concrete evidence is lacking about the true identity of bladder cancer stem cells (CSCs), there are stem-cell-like behaviors displayed by MIBC cells [12]. For instance, cell lines derived from human MIBC can undergo sphere formation in serum-free and low-adherent culture conditions [13]. Further, human MIBC can be serially transplanted in xenografts [14,15]. MIBC is highly heterogeneous, comprised of cellular lineages as diverse as urothelial, squamous, and neuronal [8,16,17,18], presumably due to divergent differentiation from common ancestral CSCs. Biologically aggressive MIBC cells also tend to exhibit features of epithelial–mesenchymal transition (EMT) [19,20]. Furthermore, the great majority of MIBCs are either intrinsically resistant to platinum-based chemotherapeutics or acquire drug resistance during the course of platinum therapy [21]. Taken together, the stemness of bladder cancer cells contributes in large measure to the genesis and progression of MIBC. Clearly, elucidating the mechanisms underlying the stemness and invasion of bladder cancer cells has the potential to open new avenues for treatment strategies. Nevertheless, despite some recent attempts, this is an area where significant knowledge gaps remain.

One topic that has attracted growing recent attention relates to the importance of long non-coding RNA (lncRNA) in regulating cancer-cell-like behaviors, including stemness and invasion [22,23]. Small nucleolar RNA host gene 1, or *SNHG1*, is an lncRNA that is over-expressed in a broad range of cancer types [24]. *SNHG1* is believed to exert oncogenic effects by inhibiting tumor suppressors, such as Tp53, and by affecting the activities of certain miRNA that regulate tumorigenic processes, such as cell proliferation, migration, apoptosis, and EMT [25]. The over-expression of *SNHG1* is considered a marker of progression for non-small-cell lung [26], cervical [26], and pancreatic cancers [27]. However, very little is known about whether *SNHG1* participates in the stem-cell-like and invasive properties of cancer cells and, if so, what signaling pathway conducts such effects.

We carried out the present study to determine the functional significance of the preferential over-expression of *SNHG1* in advanced human bladder cancers. We examined how the enforced over-expression, and conversely the knockdown, of *SNHG1* affected bladder cancer cell sphere formation, migration, and invasion. We screened for, pinpointed, and functionally validated specific signaling molecules and mechanisms of action of key components of the *SNHG1* effector pathway that stimulated bladder cancer cell stemness and invasion. In addition, we studied the functional relationship between stemness, migration, and invasion. By mining the existing human bladder cancer databases, we found that the high levels of expression of several activators within our identified *SNHG1* effector pathway were strongly associated with poor patient survival; these activators may serve as actionable targets during therapies to control bladder cancer invasion and metastasis.

## 2. Materials and Methods

### 2.1. Reagents, Antibodies, and Plasmids

The following reagents were obtained commercially: actinomycin D from Santa Cruz, a dual luciferase assay kit from Promega, and TRIzol and the SuperScript™ First-Strand Synthesis system from Invitrogen. Full-length human *SNHG1* sequences were synthesized and subcloned into pmR-ZsGreen1 (Takara Bio, Shiga, Japan). The DNMT3A knockout plasmid and the *miR-129-2* expression plasmid were obtained from Addgene. The 3′-UTR of *Rac1* mRNA was cloned into the p-MIR luciferase reporter vector. The primary antibodies against SOX2 (3579S), OCT4 (2840S), and CD133 (64326S) were purchased from Cell Signaling; the antibodies against DNMT3A (sc-373905), DNMT1 (sc-271729), and Rac1 (sc-95) from Santa Cruz; the antibody against β-Actin (A5441) from Sigma-Aldrich; and the antibody against DNMT3B (GTX129127) from Gentex.

### 2.2. Cell Lines, Culture, and Transfection

The human bladder cancer cell line T24T, a derivative of the T24 cell line, was a kind gift from Dr. Dan Theodorescu of the Cedars-Sinai Cancer Center. UMUC3 was purchased from ATCC. Both cell lines were cultured in DMEM: F-12 (1:1) with 5% FBS (Atlanta Biologicals, Flowery Branch, GA, USA) [28,29]. The cell lines were authenticated every 6 months by testing the STR loci and gender using the PowerPlex^®^ 16 HS System (Genetica DNA Laboratories, Cincinnati, OH, USA). Results were compared with data from the ATCC STR Database. Cell transfection was performed using PolyJetTM DNA (SL10068) In Vitro Transfection Reagent (SignaGen Laboratories, Frederick, MD, USA). For stable transfection, cells were selected with various antibiotics based on the specific constructs involved. The cells that survived the selection were pooled as mass stable transfectants.

### 2.3. Sphere Formation Assay

Cultured cells were plated into 6-well ultralow attachment plates (Corning, Corning, NY, USA) at 1000 cells/well in 2 mL of DMEM/F-12 supplemented with 10% FBS. The number of spheres was counted at day 7 after seeding, and the results were tabulated relative to the control groups.

### 2.4. Cell Invasion Assay

Upon incubation with the Transwell (BD Falcon, Schaffhausen, Switzerland), the cells on both interior sides of the chamber were fixed in 3.7% formalin for 2 min and 100% methanol for 20 min and stained with Giemsa. The non-invading cells were scraped off with a cotton swab, and the invaded cells were normalized to migrated cells. Cell migration was assessed in Transwell without Matrigel. The images were captured under an Olympus DP71, and the number of cells was calculated using NIH ImageJ software.

### 2.5. Luciferase Reporter Assay

The UCSC Genome Browser was used to search for the promoter region and 3′-UTR in genes of interest. TargetScan was used to search for potential miRNA binding sites in 3′-UTR of *Rac1* mRNA. The promoter-driven and 3′-UTR-driven luciferase reporters were then constructed and transfected together with the Renilla luciferase vector pRL-TK (Promega, Madison, WI, USA) into cultured bladder cancer cells. The luciferase activity was determined by using the dual-luciferase assay kit (Promega) together with a luminometer (Lumat). The firefly luciferase signal was normalized to the Renilla luciferase signal to control the variability in transfection efficiencies.

### 2.6. Immunoblotting Analysis

Cultured cells were lysed in a cell-lysis buffer (10 mm Tris-HCl (pH 7.4), 1% SDS, 1 mm Na3VO4, and a cocktail of proteinase inhibitors). The protein concentration was determined using Nano Drop 2000 (Thermo Scientific, Waltham, MA, USA). The protein extracts were subjected to Western blotting using the primary antibodies described above. The antibody-reactive protein bands were visualized using an alkaline-phosphatase-linked secondary antibody and an enhanced chemifluorescence system (Amersham Biosciences, Amersham, UK). The relative abundance of the proteins was semi-quantified by scanning the bands with the NIH ImageJ software and expressed as a ratio against the identically quantified β-actin control. The results represented at least three independent experiments.

### 2.7. Chromatin Immunoprecipitation (ChIP) Assay

MethPrimers were used to analyze CpG islands of the *miR-129-2* promoter, which served as potential binding sites for DNMT3A. The EZ-ChIP kit (Millipore Technologies) was used to carry out the ChIP assay. Briefly, the T24T cells were treated with 1% formaldehyde for 10 min at room temperature. The cells were then pelleted, resuspended in lysis buffer, and sonicated to generate 200–400 bp chromatin DNA fragments. After centrifugation at 13,000× *g* at 4 °C for 10 min, the supernatants were incubated with a rabbit anti-DNMT3A antibody or non-immune rabbit control IgG at 4 °C overnight. The immune complex was captured with Protein-G-agarose-saturated beads with salmon sperm DNA and then eluted with elution buffer. The reverse cross-linking of protein–DNA complexes to free DNA was conducted by incubation at 65 °C overnight. The DNA was extracted and subjected to real-time PCR analysis.

### 2.8. RNA Immunoprecipitation (RIP)

The cultured cells were lysed in a polysome lysis buffer containing 10 mM HEPES pH 7.0, 100 mM KCl, 5 mM MgCl_2_, 25 mM EDTA, 0.5% IGEPAL, 2 mm DTT, 50 units/mL RNase OUT, 50 units/mL Superase IN, 0.2 mg/mL heparin, and a complete proteinase inhibitor. The lysates were centrifuged at 14,000× *g* for 10 min at 4 °C. The anti-DNMT3A antibody and agarose beads A/G were added to the supernatants and incubated overnight at 4 °C in a NET2 buffer containing 50 mM Tris-HCl, pH 7.4, 150 mM NaCl, 1 mM MgCl_2_, 0.05% IGEPAL, 50 units/mL Rnase OUT, 50 units/mL Superase IN, 1 mM dithiothreitol, and 30 mM EDTA. The agarose beads were washed three times, resuspended in 100 μL of NET2 and 100 μL of SDS-TE (20 mM Tris-HCl, pH 7.5, 2 mM EDTA, and 2% SDS), and then incubated for 30 min at 55 °C. The RNAs were extracted by phenol-chloroform-isoamyl alcohol, after which quantitative PCR was performed to detect *SNHG1* present in the immune complex.

### 2.9. Quantitative Real-Time PCR

Total RNAs were extracted with TRIzol (Invitrogen) and reverse-transcribed to cDNAs using the Thermo-Script RT-PCR system (Invitrogen). A pair of oligonucleotides (forward: 5′-gatgatcttgaggctgttgtc-3′ and reverse: 5′-cagggctgcttttaactctg-3′) was used to amplify human GAPDH cDNA as an internal control. The human *SNHG1* cDNA fragments were amplified using primer pairs: 5′-agcagacacagattaagaca-3′ (forward) and 5′-ggcaggtagattccagataa-3′ (reverse); the human Rac1 cDNA fragments were amplified using primer pairs: 5′-atcaagtgtgtggtggtggg-3′ (forward) and 5′-ccagctgtatcccataagccca-3′ (reverse). Total microRNAs were extracted using the miRNeasy Mini Kit (QIAGEN, Germantown, MD, USA), reverse transcription was performed using the miScript II RT Kit (QIAGEN), and quantitative PCR was performed using the miScript PCR Starter Kit (QIAGEN). U6 was used as an endogenous normalizer. Cycle threshold (CT) values were determined, and the relative expression of microRNAs was calculated using the values of 2-^ΔΔCt^.

### 2.10. DNA Extraction, Bisulfite DNA Modification, and Methylation-Specific PCR

Genomic DNA was extracted using the Dneasy Blood & Tissue Kit (Qiagen). The sodium bisulfite modification of DNA and the subsequent isolation of the sodium bisulfite conversion of unmethylated cytosine in DNA were performed using the EpiTect Bisulfite kit (Qiagen). Optimized methylation-specific PCR was employed to amplify bisulfite-treated genomic DNA [30]. The primers of *miR-129-2* MSP were: M-Forward: 5′-TTTTAGTTCGTATTAATGAGTTGGC-3′; M-Reverse: 5′-CGAATCTCTAAACAAATACAATTCGA-3′; U-Forward: 5′-TTAGTTTGTATTAATGAGTTGGTGG-3′; U-Reverse: 5′-AAATCTCTAAACAAATACAATTCAAA-3′.

### 2.11. RNA Pull-Down

*SNHG1* was in vitro transcribed and biotin-labeled using a biotin RNA labeling mix (Roche, Basel, Switzerland). An antisense RNA sequence of *SNHG1* was used as negative control. Biotin-labeled products of *SNHG1* and its antisense controls were treated with RnasE–Free Dnase I (Roche, Basel, Switzerland) and purified with the Rneasy Mini Kit (Qiagen). UMUC3 cell extracts were incubated with biotin-labeled products at 4 °C for 1 h, followed by the addition of streptavidin agarose beads (Invitrogen, Waltham, MA, USA). DNMT3A protein in each reaction was detected by Western blotting.

### 2.12. Statistical Analysis

Two-sided Student’s *t*-tests were used to determine significant differences between the experimental groups and the control groups, and a *p*-value < 0.05 was considered significant.

## 3. Results

### 3.1. SNHG1 Over-Expression Induces the Stem-Cell-like and Invasive Behaviors of Cultured Bladder Cancer Cells by Upregulating Rac1 Expression

**Figure 1:**
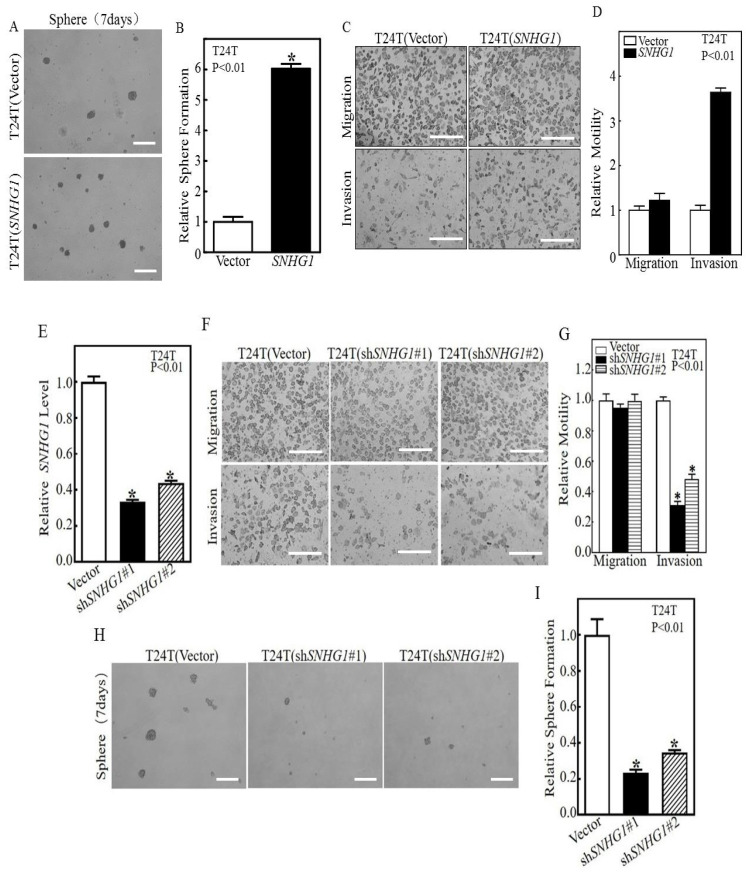
The over-expression of *SNHG1* induced stem-cell-like sphere formation and invasion; conversely, the knockdown of *SNHG1* inhibited sphere formation and invasion. (**A**,**B**) T24T cells stably transfected with a mock vector (T24T(Vector)) or *SNHG1* (T24T(*SNHG1*)) were plated into ultra-low attachment plates. After 7 days of culture, representative images of sphere colonies were photographed (**A**), and the number of tumor spheres was counted and presented relative to T24T(Vector) cells (**B**). (**C**,**D**) The migration and invasion of T24T(Vector) and T24T(*SNHG1*) cells were both determined using a BD BiocoatTM MatrigelTM invasion chamber. Migration was determined using the empty insert membrane without Matrigel, while invasion was evaluated using the same system except that Matrigel was applied (**C**). The invasiveness was normalized to the insert control according to the manufacturer’s instructions (**B**). (**E**) T24T cells were stably transfected with a mock vector (T24T(Vector)) or two independent shRNAs of *SNHG1*, i.e., T24T(sh*SNHG1*#1) or sh*SNHG1*#2. The knockdown of *SNHG1* is indicated with an asterisk (*) indicating significant decreases in comparison to T24T(Vector) cells (*p* < 0.01). (**F**,**G**) The stable transfectants were assessed for their migration and invasion with the same methods as with *SNHG1* over-expression. (**H**,**I**) T24T(Vector), T24T(sh*SNHG1*#1), and T24T(sh*SNHG1*#2) cells were plated into ultra-low attachment plates. After 7 days of culture, representative images of sphere-forming colonies were captured under microscopy (**H**), and the number of spheres formed was counted and presented relative to T24T(Vector) cells (**B**). Bars in (**A**,**H**) are equal to 200 μM and equal to 100 μm in (**C**,**F**).

To determine the functional significance of SNHG1, which is frequently over-expressed in human MIBC specimens (Figure S1A), we stably transfected cultured T24T bladder cancer cells with a SNHG1-bearing plasmid. Stable transfectants bearing the SNHG1 plasmid, i.e., T24T(SNHG1), expressed significantly higher levels of SNHG1 than stable transfectants bearing the vector control, i.e., T24T(Vector) (Figure S1B). We next compared these two transfectants for their ability to form stem-cell-like spheres and found that the T24T(SNHG1) cells formed nearly six times more spheres than the T24T(Vector) cells (Figure 1A,B). Additionally, while T24T(SNHG1) cells and T24T(Vector) cells showed little difference in cell migration, the former exhibited over three-fold more invasion than the latter (Figure 1C,D). To complement the over-expression experiments, we performed the shRNA knockdown of SNHG1 in T24T cells (Figure 1E) and found that the two shRNAs located in different regions of SNHG1 (Table S1) caused a significant reduction in cell invasion without a significant change in cell migration (Figure 1F,G). Knocking down SNHG1 also led to a marked reduction in stem-cell-like sphere formation in T24T cells (Figure 1H,I).

**Figure 2:**
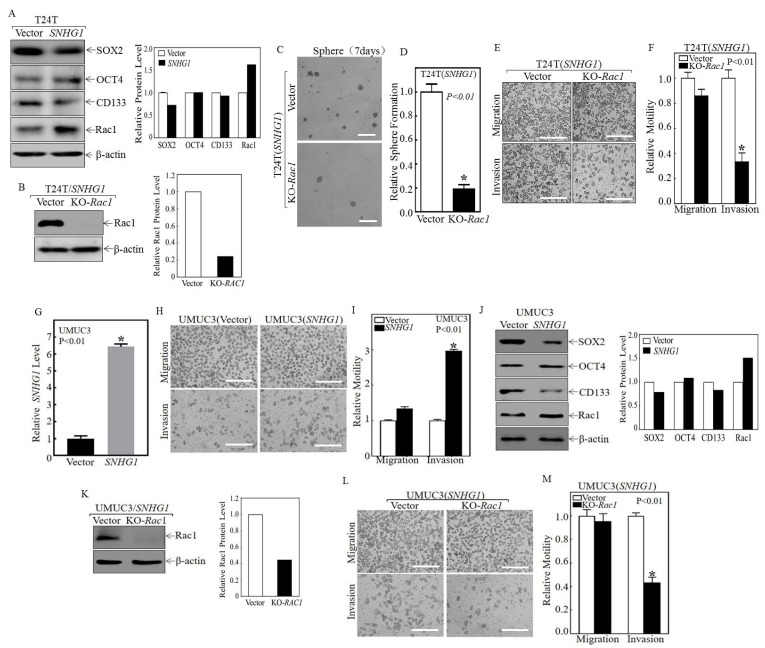
SNHG1-induced stem-cell-like and invasive behaviors were mediated by the upregulation of Rac1 in both T24T and UMUC3 cells. (**A**, Left panel) Total protein extracts from cultured T24T(Vector) and T24T(SNHG1) cells were subjected to Western blotting using antibodies against SOX2, OCT4, CD133, and Rac1. β-Actin served as a loading control. (**A**, Right panel) Relative protein levels determined by densitometry and expressed as ratios versus β-Actin. The uncropped blots and molecular weight markers of this figure and all other figures in this paper are shown in a Supplementary File S1 named “uncropped gel images”. (**B**, Left panel) CRISPR/Cas9-Rac1 or its scramble vector was stably transfected into T24T(SNHG1) cells, and the transfectants were assessed by Western blotting for Rac1 expression. β-Actin was used as a protein loading control. (**B**, Right panel) Relative protein levels determined by densitometry and expressed as ratios versus β-Actin. (**C**,**D**) The indicated cells were subjected to a stem-cell-like sphere formation assay. Representative images were photographed (**C**), and the number of spheres was counted and presented relative to T24T(SNHG1/Vector) cells (**D**). (**E**,**F**) T24T(SNHG1) cells and T24T(SNHG1/KO-Rac1) cells were subjected to transwell migration/invasion assays (**E**), and the extent of migration and invasion was calculated and presented in terms of relative motility to the T24T(SNHG1) control (**F**). The results are presented as means  ±  SD from triplicate experiments, and an asterisk (*) indicates a significant decrease in comparison to T24T(SNHG1) cells (*p*  <  0.01). (**G**) SNHG1 over-expression was verified in UMUC3(SNHG1) in comparison to UMUC3(Vector) cells, with significant increases marked with an asterisk (*) (*p* < 0.01). (**H**,**I**) Invasion of UMUC3(Vector) and UMUC3(SNHG1) cells was determined using a BD BiocoatTM MatrigelTM invasion chamber. (**J**, Left panel) The protein extracts from the indicated cells were subjected to Western blotting to determine the expression of SOX2, OCT4, CD133, and Rac1. β-Actin was used as a protein loading control. (**J**, Right panel) Relative protein levels determined by densitometry and expressed as ratios versus β-Actin. (**K**, Left panel) CRISPR/Cas9-Rac1 and its scramble vector were stably transfected into UMUC3(SNHG1) cells, and the transfectants were identified by Western blotting. (**K**, Right panel) Relative protein levels determined by densitometry and expressed as ratios versus β-Actin. (**L**,**M**) UMUC3(SNHG1) cells and UMUC3(SNHG1/KO-RAC1) cells were subjected to a transwell migration/invasion assay; the invasion rate between the cells was calculated as above. The results are presented as mean  ±  SD from triplicate experiments, and asterisks (*) indicate a significant decrease in comparison to UMUC3(SNHG1) cells (*p*  <  0.01). Bars in (**C**) are equal to 200 μM and equal to 100 μm in (**E**,**H**,**L**).

To discern the molecular underpinnings of our observed changes in stemness and invasion due to altered SNHG1 expression, we performed Western blotting using antibodies against several proteins implicated in cancer cell stemness and invasion, including SOX2, OCT4, CD133, and Rac1 [31,32,33,34]. Among these proteins, the upregulation of Rac1 (3–5-fold induction) emerged as the most significant change in SNHG1-over-expressing cells (Figure 2A), raising the possibility that Rac1 may act downstream of SNHG1 to promote stemness and invasion. To explore this possibility, we knocked out Rac1 in T24T(SNHG1) cells using the CRISPR/cas9 approach (Figure 2B). T24T(SNHG1) cells lacking Rac1 had a marked decrease in sphere formation (Figure 2C,D) and invasion, without significant changes in migration (Figure 2E,F), compared to T24T(SNHG1) cells transfected with a vector harboring a scrambled gRNA. These effects were reproduced in an independent human bladder cancer cell line, UMUC3 (Figure 2G–M). I enforced over-expression of SNHG1 promoted invasion (Figure 2G–I) by upregulating Rac1 but not other known stem cell signaling molecules (Figure 2J); in addition, downregulating Rac1 in SNHG1-over-expressing cells inhibited cell invasion (Figure 2K–M). In addition, notably, in the TCGA database of human MIBC, Rac1 mRNA is significantly over-expressed in MIBC specimens compared to normal bladder controls (Figure S2), and in the Human Protein Atlas database, the high-expression group of Rac1 mRNA is more strongly associated with poor survival than the low-expression group of Rac1 (Figure S3). Taken together, these results strongly suggest that SNHG1 over-expression triggers Rac1 protein over-expression, that Rac1 induces stem-like sphere formation and invasion, and that the upregulation of Rac1 by SNHG1 promotes MIBC formation and poor prognosis.

### 3.2. SNHG1 Stabilizes Rac1 mRNA by Suppressing miR-129-2-5p Transcription

**Figure 3:**
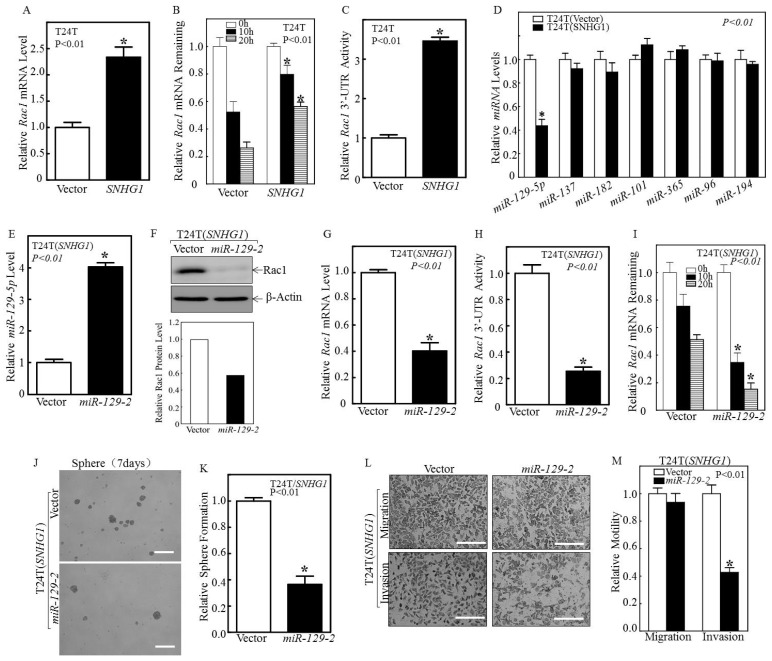
SNHG1 over-expression promoted the stability of Rac1 mRNA by inhibiting miR-129-2-5p induction in BC cells. (**A**) The Rac1 mRNA levels were quantified in T24T(Vector) and T24T(SNHG1) cells by real-time PCR with GAPDH as an internal control. (**B**) The T24T(Vector) and T24T(SNHG1) cells were seeded into 6-well plates; after synchronization, the cells were treated with Act-D for 0, 10, and 20h. Total RNA was then isolated and subjected to the real-time PCR quantification of Rac1 mRNA with GAPDH as an internal control. (**C**) The Rac1 mRNA 3′-UTR luciferase reporter was transiently transfected into the indicated cells, and the luciferase activity of each transfectant was assayed and normalized using pRL-TK as an internal control and presented relative to the vector transfectant. (**D**) Quantitative real-time PCR was carried out to determine the expression levels of key miRNAs in the indicated cells. (**E**) Stable transfection of miR-129-2 plasmids into T24T(SNHG1) cells, followed by the detection of miR-129-5p by real-time PCR. (**F**, Top panel) Protein lysates extracted from the indicated cells were subjected to Western blotting to assess Rac1 expression. β-Actin served as a loading control. (**F**, Bottom panel) Relative protein levels determined by densitometry and expressed as ratios versus β-Actin. (**G**) Quantitative real-time PCR was carried out to determine the expression of Rac1 mRNA in the indicated cells. (**H**) Wild-type Rac1 3′-UTR mRNA luciferase reporters were transiently co-transfected with pRL-TK into the indicated cells. The luciferase activity of each transfectant was evaluated, and the results are presented relative to Rac1 3′-UTR activity. (**I**) T24T(SNHG1) and T24T(SNHG1/miR-129-2) cells were incubated with Act-D for 0, 10, and 20h. Total RNA was isolated, and quantitative real-time PCR was then performed to determine Rac1 mRNA levels. The results are presented as means  ±  SD from triplicate experiments, and asterisks (*) indicate a significant difference in comparison to T24T(SNHG1) cells (*p*  <  0.01). (**J**,**K**) T24T(SNHG1) and T24T(SNHG1/miR-129-2) cells were seeded into ultra-low attachment plates to determine sphere formation. Seven days later, the representative images of spheres were captured under microscopy (**J**), and the number of spheres was counted and presented relative to T24T(SNHG1) cells (**K**). (**L**,**M**) T24T(SNHG1) cells and T24T(SNHG1/miR-129-2) cells were subjected to a transwell invasion assay (**L**), and migration and invasion were calculated and presented relative to the T24T(SNHG1) control. The results are presented as means  ±  SD from triplicate experiments, and asterisks (*) indicate a significant decrease in comparison to T24T(SNHG1) cells (*p*  <  0.01) (**M**). Bars in (**J**) are equal to 200 μM and equal to 100 μm in (**L**).

To ascertain the molecular basis by which SNHG1 upregulates Rac1, we first assessed the levels of Rac1 mRNA in the T24T(SNHG1) cells versus the T24T(Vector) cells. Rac1 mRNA was markedly upregulated in T24T(SNHG1) cells compared to T24(vector) cells (Figure 3A). To distinguish whether the upregulation of Rac1 mRNA by SNHG1 was due to mRNA over-expression or increased stability, we assessed Rac1 mRNA stability in the presence of actinomycin D (or Act-D) to prevent de novo mRNA synthesis. Rac1 mRNA was much more stable in the T24T(SNHG1) cells than in the T24T(Vector) cells (Figure 3B). Given the fact that the 3′-UTR of mRNA affects mRNA stability [35], we tested this possibility and found that SNHG1 significantly promoted Rac1 mRNA 3′-UTR activity (Figure 3C). Since microRNAs act as the key regulators in mRNA stability by binding to the 3′-UTR of target genes to affect mRNA stability [36], we used TargetScan to search for the potential miRNA binding sites within the 3′-UTR of Rac1 mRNA and their corresponding miRNAs (Table S1). Based on this survey, we performed real-time PCR to quantify the relative expression levels of the candidate miRNAs in T24T(Vector) and T24T(SNHG1) cells. We found that miR-129-5p, but not any other of this set of miRNAs, was specifically downregulated in SNHG1-over-expressing T24T cells (Figure 3D). Consistent with this observation, we found that the over-expression of SNHG1 also inhibited miR-129, and the knockdown of SNHG1 increased miR-129 in UMUC3 cells (Figure S5). Based on these results, we anticipated that miR-129-5p might be involved in the SNHG1 regulation of the 3′-UTR activity of Rac1 mRNA. To test this, we stably over-expressed miR-129-2, the key precursor of miR-129-5p in T24T(SNHG1) cells and UMUC3(SNHG1) (Figure 3E and Figure S4B). The over-expression of miR-129-2 completely blocked the SNHG1-mediated induction of Rac1 protein and mRNA (Figure 3F,G and Figure S4C). Additionally, miR-129-2 over-expression markedly decreased 3′-UTR activity (Figure 3H) and the stability of Rac1 mRNA (Figure 3I). Furthermore, miR-129-2 over-expression blocked the SNHG1-mediated stem-cell-like sphere formation and invasion of MIBC cells (Figure 3J–M and Figure S3D,E). Finally, the knockdown of SNHG1 markedly reduced Rac1 expression, which closely correlated with miR-129-5p over-expression (Figure S4A,B). Altogether, we clearly show that SNHG1 over-expression inhibits miR-129-5p expression, thereby reducing the ability of the latter to bind to the 3′-UTR Rac1 mRNA and increasing the stability of Rac1 mRNA and promoting bladder cancer cell stemness and invasion.

### 3.3. SNHG1 Binds DNMT3A, and the Binding Tethers DNMT3A to the miR-129-2 Promoter, Hyper-Methylates It, and Suppresses Its Transcription

**Figure 4:**
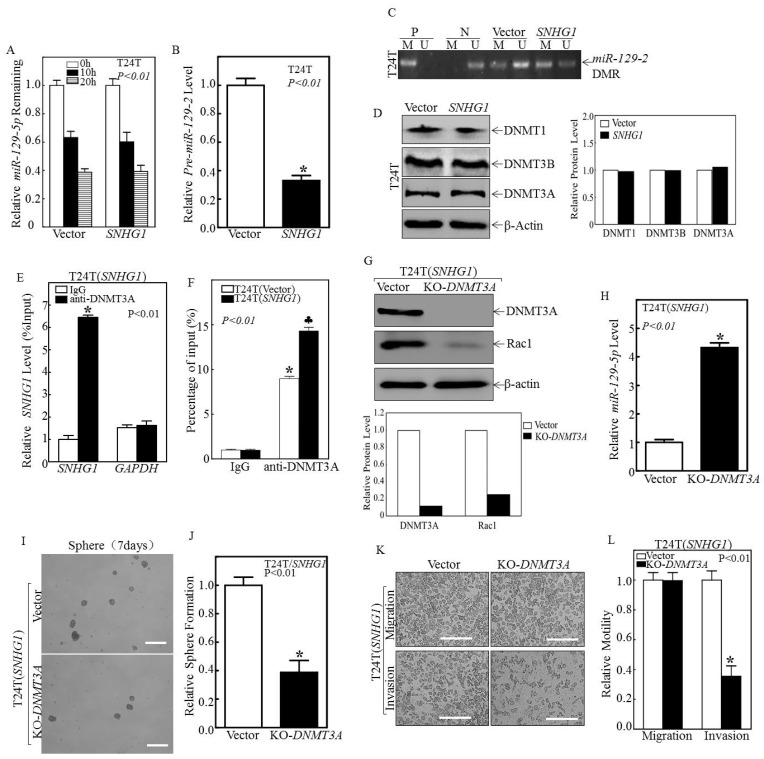
SNHG1 bound to DNMT3A protein and promoted the interaction of DNMT3A with the miR-129-2 promoter, thereby hyper-methylating and inhibiting the transcription of miR-129-2. (**A**) T24T(Vector) and T24T(SNHG1) cells were incubated with Act-D for 0, 10, and 20h. Total RNA was isolated and then subjected to quantitative real-time PCR to determine miR-129-5p levels. (**B**) Quantitative real-time PCR was carried out to determine the expression of pre-miR-129-2 in the indicated cells. (**C**) The methylation status of the miR-129-2 promoter in the differentially methylated region (DMR) was determined using a methylation-specific PCR (MS-PCR) assay. Two primer sets were used to evaluate the methylated (M) and unmethylated (U) copies of the miR-129-2 DMR gene. Methylated DNA was used as the positive control (P), while unmethylated DNA was used as the negative control (N). PCR products represented the methylated state and unmethylated allele. (**D**, Left panel) Total protein extracts from the indicated cells were extracted and subjected to Western blotting for DNMT1, DNMT3A, and DNMT3B, with β-Actin as a loading control. (**D**, Right panel) Relative protein levels determined by densitometry and expressed as ratios versus β-Actin. (**E**) RNA-IP was carried out to examine the specific interaction of SNHG1 with DNMT3A. GAPDH was employed as a negative control. (**F**) ChIP assays were performed to determine DNMT3A binding to the miR-129-2 promoter in T24T(Vector) and T24T(SNHG1) cells (see Materials and Methods for details). (**G**,**H**) CRISPR/Cas9 specifically targeting DNMT3A was stably transfected into T24T(SNHG1) cells. The cell lysates were subjected to Western blotting to determine DNMT3A and Rac1 protein expression (**G**), and the isolated RNAs were subjected to quantitative real-time PCR to evaluate miR-129-5p levels (**H**). (**I**,**J**) The indicated cells were subjected to a stem-like sphere formation assay. Representative images of formed spheres were captured under microscopy (**I**), and the number of spheres f was counted and presented as sphere formation relative to T24T(SNHG1/Vector) cells (**J**). (**K**,**L**) T24T(SNHG1/Vector) cells and T24T(SNHG1/KO-DNMT3A) cells were subjected to a transwell invasion assay, and migration and invasion abilities were calculated and presented in terms of relative motility to the T24T(SNHG1/Vector) control. The results are presented as means  ±  SD from at least triplicate experiments, and asterisks (*) indicate a significant decrease in comparison to T24T(SNHG1/Vector) cells (*p*  <  0.01). Bars in (**I**) are equal to 200 μM and equal to 100 μm in (**K**).

**Figure 5:**
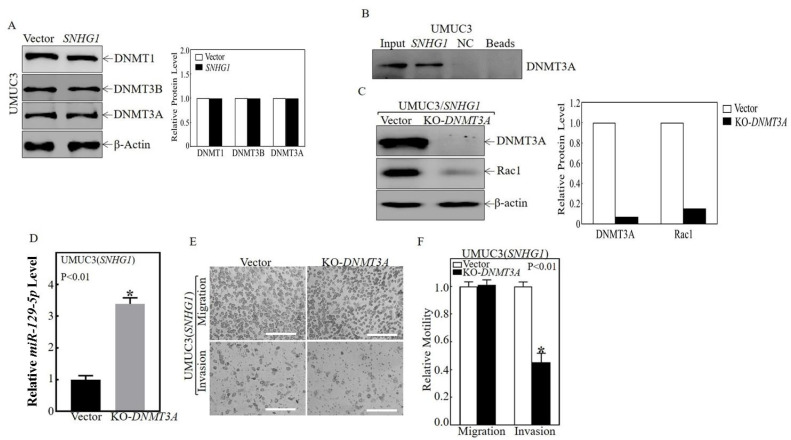
The binding of SNHG1 to DNMT3A protein inhibited miR-129-5p expression and increased Rac1 expression in UMUC3 cells. (**A**, Left panel) The total protein extracts from indicated cells were subjected to Western blotting to determine the expression of DNMT1, DNMT3A, and DNMT3B. (**A**, Right panel) Relative protein levels determined by densitometry and expressed as ratios versus β-Actin. (**B**) An RNA pull-down assay was employed to determine the interaction between SNHG1 and DNMT3A. Antisense RNA sequences of SNHG1 were used as negative controls (NC). (**C**, Left panel) The indicated cells were extracted, and protein extracts were subjected to Western blotting to determine DNMT3A, Rac1, and β-Actin protein expression. (**C**, Right panel) Relative protein levels determined by densitometry and expressed as ratios versus β-Actin. (**D**) Quantitative real-time PCR was carried out to determine the expression levels of miR-129-5p in the indicated cells. (**E**,**F**) UMUC3(SNHG1) cells and UMUC3(SNHG1/KO-DNMT3A) cells were subjected to a transwell invasion assay, and migration and invasion abilities were calculated and presented relative to the UMUC3(SNHG1) control. The results are presented as means  ±  SD from triplicate experiments, and asterisks (*) indicate a significant decrease in comparison to UMUC3(SNHG1) cells (*p*  <  0.01). Bars in (**E**) are equal to 100 μm.

We next sought to further investigate the mechanism(s) by which SNHG1 inhibits miR-129-5p transcription. We found that the over-expression of SNHG1 did not significantly affect the stability of miR-129-5p (Figure 4A). To examine whether SNHG1 regulated miR-129-5p at the transcriptional level, we studied the effects of SNHG1 on the expression of pre-miR-129-2. As shown in Figure 4B, as with the mature miR-129-2, the levels of pre-miR-129-2 were significantly diminished in SNHG1-over-expressing cells. Because the promoter region of miR-129-2 can be persistently hyper-methylated, leading to transcriptional inhibition in other cancer types [37], we tested whether the regulatory effects of SNHG1 on miR-129-2 could be mediated by a differentially methylated region (DMR) within the miR-129-2 promoter in MIBC cells. Upon the over-expression of SNHG1, the methylated DNA (M) increased, whereas the unmethylated DNA (U) decreased in the promoter region of miR-129-2 (Figure 4C), suggesting that SNHG1 inhibits miR-129-2 transcription via the epigenetic methylation of the miR-129-2 promoter. Since DNA methyltransferases (DNMTs) are major regulators in this process [38], we explored whether they were involved in hyper-methylating miR-129-2. As shown in Figure 4D and Figure 5A, the over-expression of SNHG1 did not affect the expression of DNMT proteins per se. Instead, our RNA immunoprecipitation assays showed that the SNHG1 transcript could be specifically precipitated by anti-DNMT3A antibody from T24T(SNHG1) cells (Figure 4E). RNA pull-down assays extended this observation, demonstrating that SNHG1 could directly bind to DNMT3A (Figure 5B). To further verify this finding, we carried out ChIP assays and found that the promoter region of miR-129-2 was specifically present in the immunoprecipitated complex pulled down by anti-DNMT3A antibody (Figure 4F). Moreover, the over-expression of SNHG1 enhanced the interaction between DNMT3A and the miR-129-2 promoter (Figure 4F; the right two columns). To functionally link DNMT3A with the SNHG1-mediated regulation of miR-129-2 and Rac1, we stably knocked out DNMT3A using CRISPR/Cas9 in T24T(SNHG1) cells (Figure 4G). This significantly inhibited Rac1 protein expression and induced miR-129-2 expression (Figure 4G,H). These effects were entirely reproducible in UMUC3 cells (Figure 5C,D). DNMT3A knockout also inhibited stem-like sphere formation and the invasive properties induced by SNHG1 over-expression (Figure 4I–L and Figure 5E,F). Taken together, these results demonstrate that SNHG1 over-expression enhanced its interaction with DNMT3A and promoted the binding of DNMT3A to the miR-129-2 promoter, resulting in the hyper-methylation of the miR-129-2 promoter and its transcriptional inhibition.

### 3.4. Sphered T24T Cells Are More Invasive than Non-Sphered Counterparts

**Figure 6:**
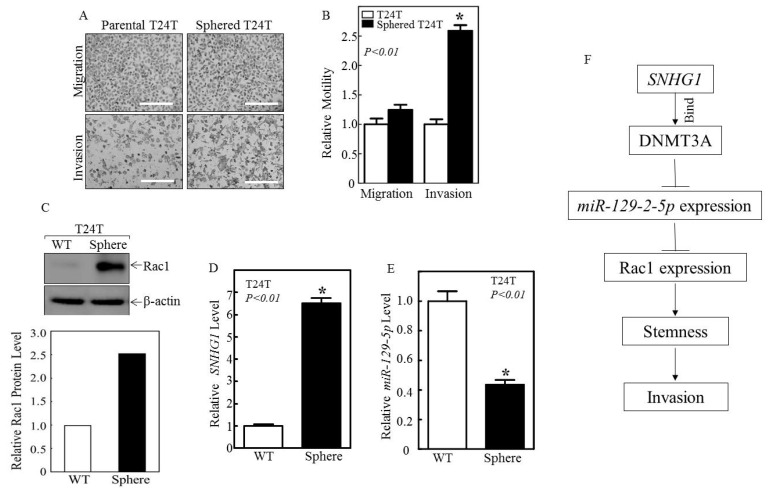
The sphere-forming T24T cells with high expression of SNHG1 and Rac1 and low expression of miR-129-5p were markedly more invasive than the non-sphere-forming T24T cells. (**A**,**B**) The isolated sphered T24T cells were subjected to transwell migration and invasion assays, and the results are presented relative to their parental T24T cells. (**C**–**E**) The parental T24T and sphered T24T cells were collected, and the proteins were extracted and subjected to either Western blotting to assess the expression of Rac1 (**C**) or real-time PCR to determine the expression of SNHG1 (**D**) and miR-129-5p levels (**E**). The results are presented as means  ±  SD from triplicate experiments, and asterisks (*) indicate a significant difference (*p*  <  0.01). (**F**) A schematic model of the SNHG1/DNMT3A/miR-129-5p/Rac1 signaling pathway in regulating the stem-cell-like and invasive properties of advanced bladder cancer cells. Bars in (**A**) are equal to 100 μm.

Having demonstrated the effects of the SNHG1/DNMT3A/miR-129-2-5p/Rac1 effector pathway on promoting bladder cancer cell stemness and invasion, we investigated more directly the functional relationship between stemness and invasion. Toward this end, we isolated sphere-forming cells from parental T24T cells and assessed their invasive ability. As shown in Figure 6A,B, T24T cells that formed spheres exhibited much higher levels of invasion than the parental T24T cells. In contrast, the ability to migrate did not differ between these two types of cells. In line with the different extents of invasion, the expression levels of Rac1 protein and SNHG1 were considerably higher in the sphere-forming T24T cells than in the parental T24T cells (Figure 6C,D). Conversely, miR-129-5p levels were significantly lower in the sphere-forming T24T cells than in the parental T24T cells (Figure 6E). These results demonstrate that SNHG1-driven stem-cell-like behavior is functionally linked to the invasive property of bladder cancer cells (Figure 6F).

## 4. Discussion

In this study, we present several key findings with significant implications for the improved understanding of the biology of muscle-invasive bladder cancer. Firstly, we found a functional link between the over-expression of *SNHG1* and the stem-cell-like behavior of bladder cancer cells. T24T cells over-expressing *SNHG1* had a markedly increased ability to form stem-cell-like tumor spheres compared to their control counterparts (Figure 1A,B), while *SNHG1* knockdown blocked this effect (Figure 1H,I). Because the stemness of cancer cells is preferentially associated with aggressive forms of cancer [10,20], our finding may provide a molecular explanation as to why *SNHG1* is over-expressed in ~95% of MIBCs but not in non-muscle-invasive bladder cancers [39]. *SNHG1*, therefore, is worthy of further evaluation as a new adjunct biomarker to predict the progression and prognosis of advanced bladder cancers [40]. Additionally, as stemness is a major contributor to tumor heterogeneity [41], *SNHG1* could be a key epigenetic regulator influencing the diverse molecular subtypes that have recently been described in bladder cancer [8,18]. Advanced bladder cancers are now known to undergo diverse forms of differentiation into different cellular lineages, including urothelial (luminal; ~65% of all muscle-invasive bladder cancers), squamous (~35%), and neuronal phenotypes (5%) [8]. The reason behind such a high degree of lineage diversity is presently unclear. Experimental evidence suggests that the altered expression of certain transcription factors, such as Foxa1, Gata3, and Pparg, can affect the molecular subtypes of bladder cancer [42]. It remains to be seen whether the over-expression of *SNHG1* simply keeps bladder cancer cells at the stem/progenitor cell state, so that they are pluripotent and capable of differentiating into different cellular lineages, or whether *SNHG1* affects bladder cancer subtypes by indirectly altering the expression of the aforementioned transcription factors.

Secondly, we found that the stemness and the invasiveness of bladder cancer cells induced by *SNHG1* over-expression are closely linked and are both driven by the over-expression of Rac1. This was evidenced by the fact that the knockdown of Rac1 by shRNA in *SNHG1*-over-expressing T24T and UMUC3 cells dramatically blocked the stimulating effects of *SNHG1* on sphere formation and invasion (Figure 2B–F,K–M). Among the common factors involved in cancer cell stemness, Rac1 stood out as the most upregulated protein in both T24T (Figure 2A) and UMUC3 cells (Figure 2J). T24T cells that formed spheres expressed markedly more *SNHG1* and Rac1 protein and were over twofold more invasive than the T24T parental cells (Figure 6A–D). Rac1 is a member of the Rac subfamily of Rho small GTPases [43] and plays a pleiotropic role in actin cytoskeleton reorganization, cell growth, cell-cell adhesion and motility, and epithelial differentiation [44]. Interestingly, Rac1 was recently shown to be important in maintaining stem cells of the normal epidermis [45] and cancer stem cells in gastric adenocarcinoma [46]. To date, however, nothing is known about the role of Rac1 in bladder cancer stemness and invasion or about the functional relationship linking Rac1 with *SNHG1*. Our findings here are, therefore, entirely novel. It should be noted that the Rac1-mediated effects on invasion and migration appeared to be separable in all our functional assays. These included the enforced over-expression of *SNHG1* (Figure 1C,D and Figure 2H,I), the knockdown of *SNHG1* by shRNA (Figure 1F,G), the knockout of Rac1 (Figure 2D–F,K–M), the enforced expression of *miR-129-2* (Figure 3L,M and Figure S4D,E; and see also later in this work), the knockout of DNMT3A (Figure 4K,L and Figure 5E,F), and the migration vs. invasion in sphered vs. non-sphered cells (Figure 6A,B). In all these experiments performed in parallel and simultaneously, only cell invasion was significantly affected. Our observation is similar to that previously reported in macrophages, where Rac1 was found to be necessary for invasion but dispensable for migration [47]. It is possible that the separable effects of Rac1 on migration and invasion are unique to *SNHG1* and/or its downstream effector pathway. Alternatively, Rac1 may specifically upregulate signals that promote invasion, such as matrix-degrading enzymes, that are not involved in cell migration [48]. Such regulatory mechanisms for invasion may even be cell-type-specific and context-dependent. These possibilities warrant further investigation.

Thirdly, by working backwards from Rac1, we identified the key signaling molecules acting between *SNHG1* and Rac1. Specifically, we found that the increased Rac1 protein in bladder cancer cells over-expressing *SNHG1* was due to the increased stability of *Rac1* mRNA (Figure 3B), and that this was associated with low levels of miR-129-5p (Figure 3D). We proved this hypothesis to be valid by over-expressing *miR-129-2*, which led to the decreased stability of *Rac1* mRNA (Figure 3I) and Rac1 downregulation (Figure 3E–G). We next identified DNMT3A, a DNA methyltransferase, as a functional link between *SNHG1* and *miR-129-2*. Through RNA immunoprecipitation, we established that *SNHG1* binds DNMT3A, and this binding leads DNMT3A to interact with, hyper-methylate, and prevent the transcription of the promoter of miR-129-5p (Figure 4C–F and Figure 5B). DNMT3A is one of the three major members of the DNA methyltransferase family, consisting of DNMT1, DNMT3A, and DNMT3B [38]. DNMT1 was over-expressed, whereas DNMT3B was downregulated in bladder cancer cells [49,50]. However, the expression status of DNMT3A in different bladder cancer variants has not been carefully assessed. Our study, to our knowledge, represents the first attempt to examine the role of DNMT3A in bladder cancer stemness and invasion. By identifying the DNMT3A/*miR-129-2-5p*/Rac1 signaling pathway downstream of *SNHG1* that is operative in advanced bladder cancer cells, we are in no way ruling out other potential pathways or components that might also mediate the activities of *SNHG1*. Global transcriptomic profiling in some of our cell lines over-expressing or downregulating *SNHG1* will be useful in addressing this question. Additionally, in vivo studies, such as xenograft (subcutaneous or orthotopic) and syngeneic models, should be helpful in further validating whether what we observed here could be extended to in vivo conditions. Some of these studies are already underway in the authors’ laboratories.

By defining a functional signaling pathway, i.e., *SNHG1*/DNMT3A/*miR-129-2-5p*/Rac1, that drives stemness and invasion in advanced bladder cancer cells, our study should spur additional investigation into the value of therapeutically targeting components of this pathway. While the technical aspects of targeting lncRNAs remain to be refined, several chemical inhibitors of Rac1 have already been developed. For instance, Rac1 Inhibitor CAS 1177865-17-6, a cell-permeable, reversible inhibitor of Rac1 GDP/GTP exchange, interferes with the interaction between Rac1 and Rac-specific GEFs [51]. More recently, Ciarlantini and colleagues identified a new family of chemical inhibitors against Rac1–GEF interaction that exhibited significant anti-proliferative activities in vitro and in a lung cancer animal model [52]. Furthermore, inhibitors have been developed that exhibited dual effects against Rac1 as well as Cdc42. These inhibitors, such as MBQ-167, have shown impressive effects in reducing tumor burden and metastasis in triple-negative breast cancer [53]. Finally, preliminary evidence suggests that Rac1 inhibitors could be combined with other target-based inhibitors to achieve synergistic effects [54]. Our search of the Human Protein Atlas database for muscle-invasive bladder cancer showed that patients with high levels of Rac1 expression faired significantly worse than patients with low levels of Rac1 expression (*p* = 0.0087; Figure S3). It would be of considerable interest to next test the various Rac1 inhibitors, first in cell-based systems and then in animal models of bladder cancer, to sort out which specific inhibitors are effective in curtailing bladder cancer growth and progression.

DNMT3A could serve as another target for inhibition because such inhibition could reduce or even reverse the hyper-methylation and silencing effects of DNMT3A on the transcription of *miR-129-2*, thus restoring the levels of *miR-129-2* and downregulating Rac1. The inhibition of DNA methylation using nucleoside analogues, such as 5-azacytidine and decitabine, has been tested as a potential therapeutic for bladder cancer [55]. As single agents, these inhibitors have yielded variable tumor-inhibitory effects, as well as toxicities that prevented their use in patients. However, it is possible that the combined use of these inhibitors with conventional chemotherapeutics and/or immune checkpoint inhibitors could reduce the dosage and, hence, the toxicities associated with the single agents; such a development would increase the effectiveness against bladder cancer cell invasion and progression. Intrinsic and acquired resistance to platinum-based chemotherapeutics is a main reason for treatment failure in managing advanced bladder cancers [56]. The stem-cell-like and invasive behaviors are major contributors to cancer heterogeneity and chemoresistance [10]. Overall, we believe that the identification of the *SNHG1*/DNMT3A/*miR-129-2-5p*/Rac1 effector pathway that plays a major role in driving bladder cancer stemness and invasion may help develop a new therapeutic approach to treat this difficult disease.

## 5. Conclusions

Although the invasion of the smooth muscle layers of the bladder is a centrally important step that fundamentally changes the outcome of patients with bladder cancer, moving them to the difficult-to-cure or incurable stage, very little is known about the molecular events driving this step. This study provides direct experimental evidence establishing the long non-coding RNA *SNHG1* as a key player in bladder cancer cell invasion. *SNHG1* does so by upregulating Rho GTPase, Rac1, in a cell-invasion-dependent but cell-migration-independent manner. Through Rac1, *SNHG1* also markedly stimulates the stem-cell-like sphere formation of bladder cancer cells. The signaling effectors downstream of *SNHG1* that trigger the invasion and stem-cell-like behaviors also involve DNMT3A and *miR-129-2-5p*. Because *SNHG1* is over-expressed in ~95% of human muscle-invasive bladder cancers, it may serve as a useful predictive marker for bladder cancer invasion and progression. Additionally, components of the *SNHG1*/DNMT3A/*miR-129-2-5p*/Rac1 signaling cascade could be targeted as therapeutic options for muscle-invasive bladder cancer, for which effective treatments remain very limited at the present time.

## Data Availability

All data generated or analyzed during this study will be included in the published article (and its Supplementary Materials).

## Supplementary Materials

The following supporting information can be downloaded at: https://www.mdpi.com/article/10.3390/cancers14174159/s1, Figure S1: Expression of *SNHG1* in human bladder cancer and enforced expression of *SNHG1* in bladder cancer cell line; Figure S2: Expression of *Rac1* mRNA in human bladder cancer; Figure S3: Rac1 mRNA expression in human bladder cancer; Figure S4: *SNHG1* over-expression inhibited *miR-129-5p* expression in UMUC3 cells; Figure S5: *SNHG1* inhibition decreased Rac1 expression and promoted *miR-129-5p* expression in UMUC3 cells; Table S1: The potential miRNA binding sites in the *Rac1* mRNA 3′UTR region; File S1: Uncropped gel images.
