# Supplementary material for: DNMT3A/miR-129-2-5p/Rac1 Is an Effector Pathway for SNHG1 to Drive Stem-Cell-like and Invasive Behaviors of Advanced Bladder Cancer Cells
Source: Cancers (Basel). 2022 Aug 27;14(17):4159. doi: 10.3390/cancers14174159 (PMC9454896; doi:10.3390/cancers14174159)
Supplement: Supplementary file 1 [file cancers-14-04159-s001.zip › cancers-1867923-File S1.pdf]

Figure2A

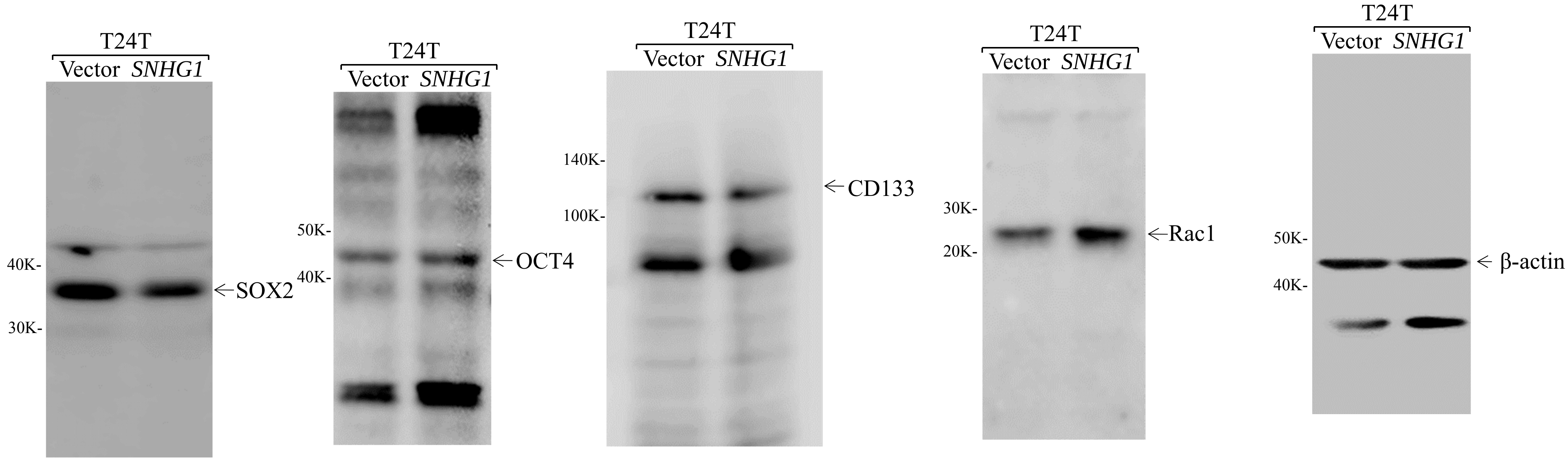

T24T
Vector SNHG1
40K-
30K-
SOX2
T24T
Vector SNHG1
50K-
40K-
OCT4
T24T
Vector SNHG1
140K-
100K-
CD133
T24T
Vector SNHG1
30K-
20K-
Rac1
T24T
Vector SNHG1
50K-
40K-
β-actin

Figure2B

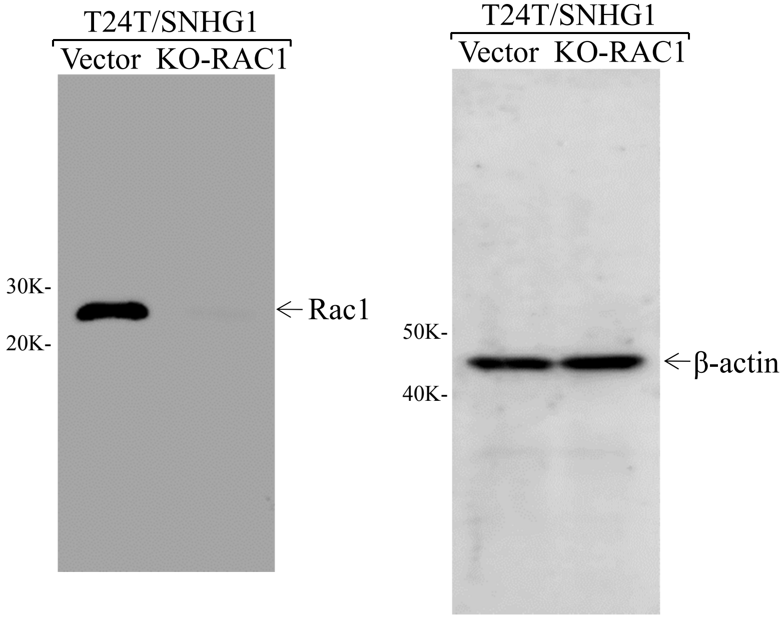
T24T/SNHG1
Vector KO-RAC1
30K-
20K-
Rac1
T24T/SNHG1
Vector KO-RAC1
50K-
40K-
β-actin

Figure2J

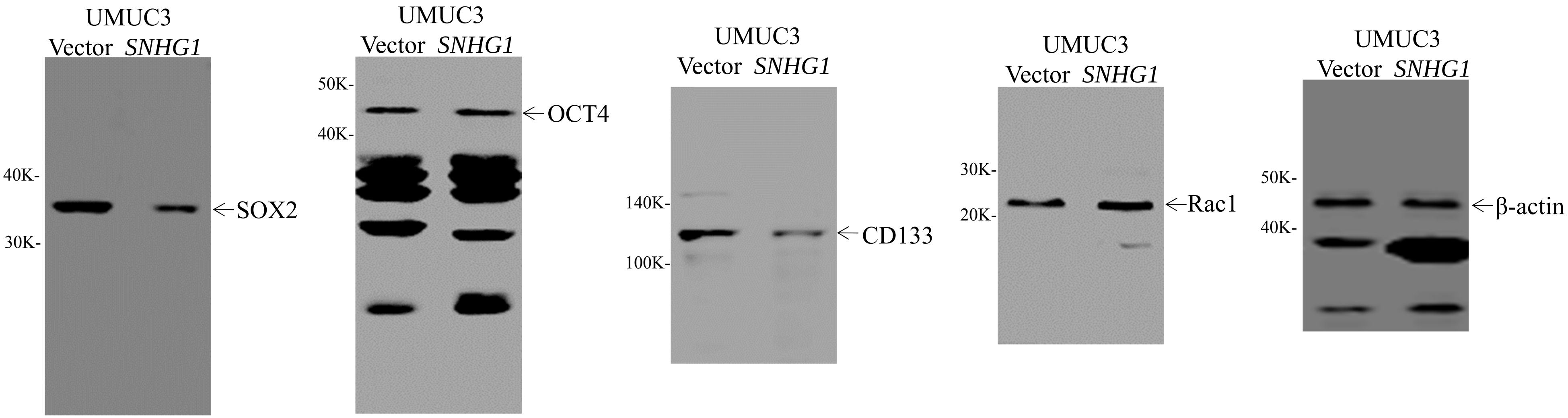

UMUC3
Vector SNHG1
40K-
30K-
SOX2
UMUC3
Vector SNHG1
50K-
40K-
OCT4
UMUC3
Vector SNHG1
140K-
100K-
CD133
UMUC3
Vector SNHG1
30K-
20K-
Rac1
UMUC3
Vector SNHG1
50K-
40K-
β-actin

Figure2K

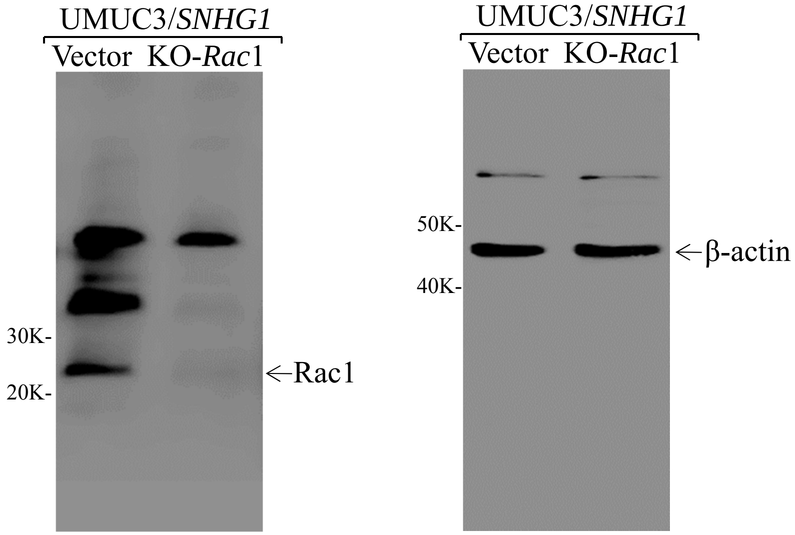
UMUC3/SNHG1
Vector
KO-Rac1
30K-
20K-
←Rac1
UMUC3/SNHG1
Vector
KO-Rac1
50K-
40K-
←β-actin

Figure3F

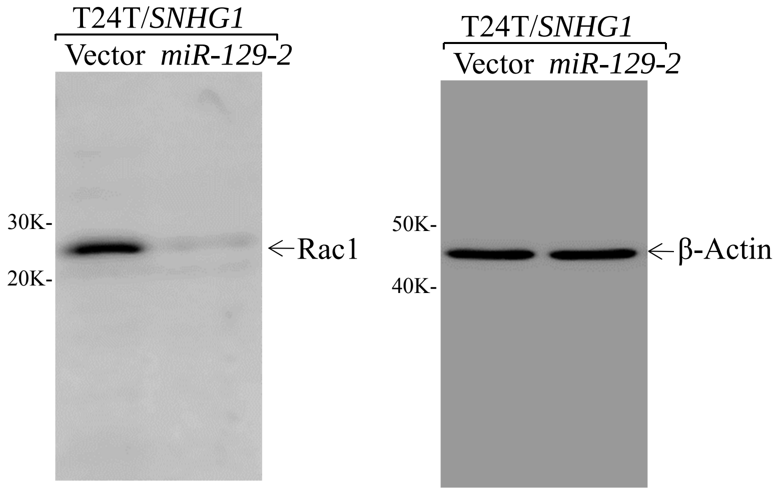
T24T/*SNHG1*
Vector *miR-129-2*
30K-
20K-
Rac1
T24T/*SNHG1*
Vector *miR-129-2*
50K-
40K-
β-Actin

Figure4D

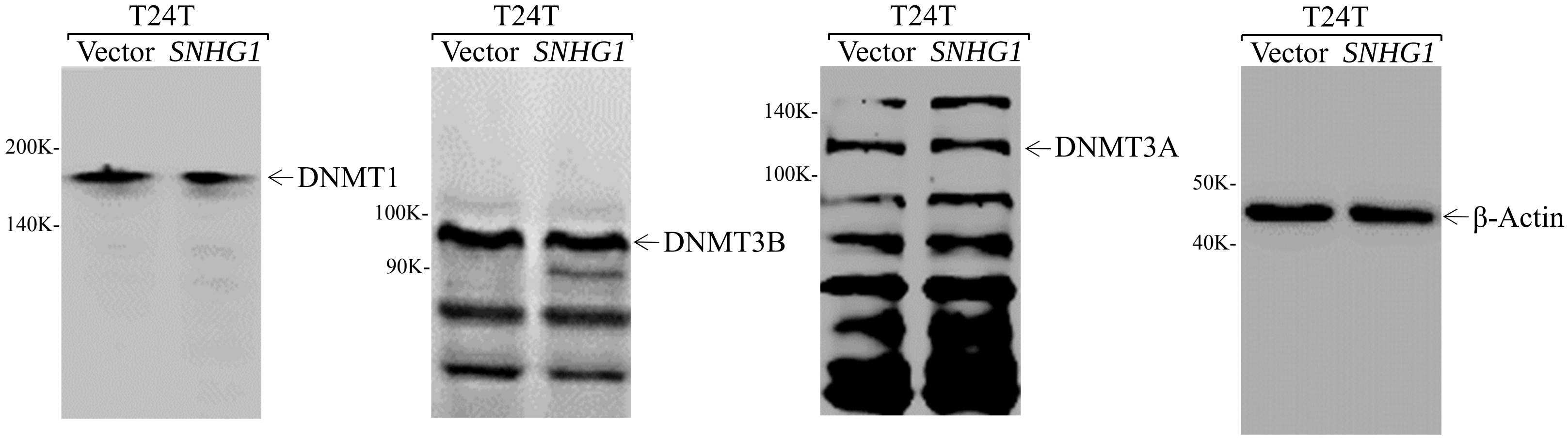
T24T
Vector
SNHG1
200K-
140K-
DNMT1
T24T
Vector
SNHG1
100K-
90K-
DNMT3B
T24T
Vector
SNHG1
140K-
100K-
DNMT3A
T24T
Vector
SNHG1
50K-
40K-
β-Actin

Figure4G

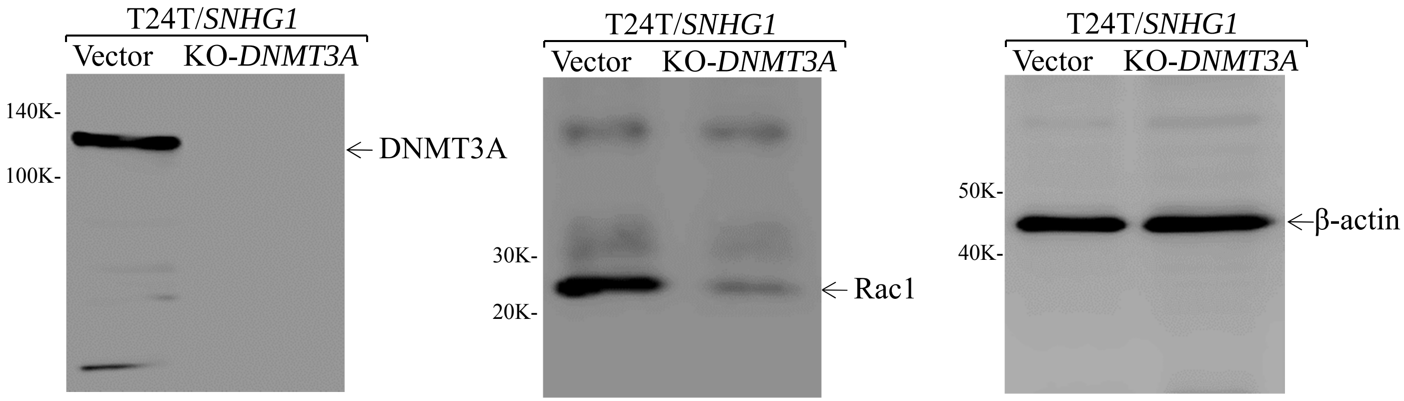
T24T/*SNHG1*
Vector
KO-*DNMT3A*
140K-
100K-
DNMT3A
T24T/*SNHG1*
Vector
KO-*DNMT3A*
30K-
20K-
Rac1
T24T/*SNHG1*
Vector
KO-*DNMT3A*
50K-
40K-
β-actin

Figure5A

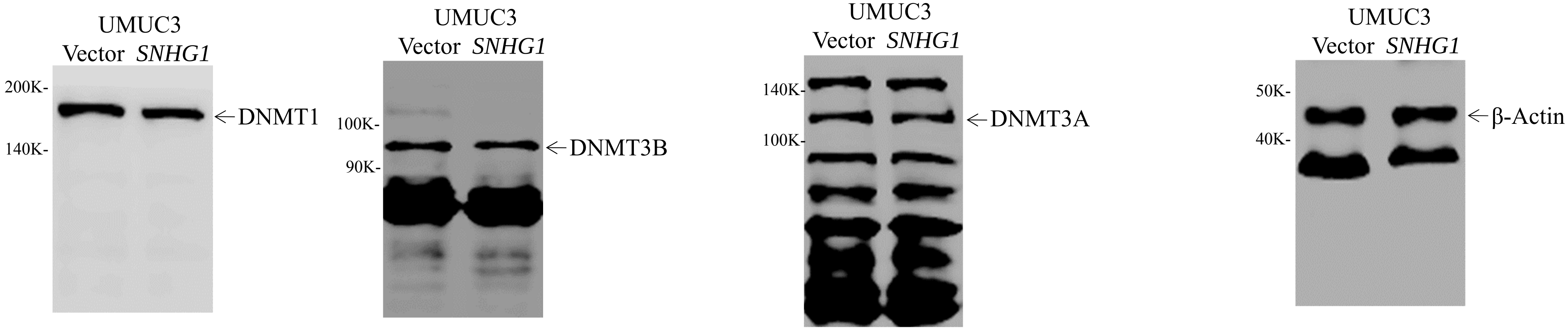
UMUC3
Vector SNHG1
200K-
140K-
DNMT1
UMUC3
Vector SNHG1
100K-
90K-
DNMT3B
UMUC3
Vector SNHG1
140K-
100K-
DNMT3A
UMUC3
Vector SNHG1
50K-
40K-
β-Actin

Figure5C

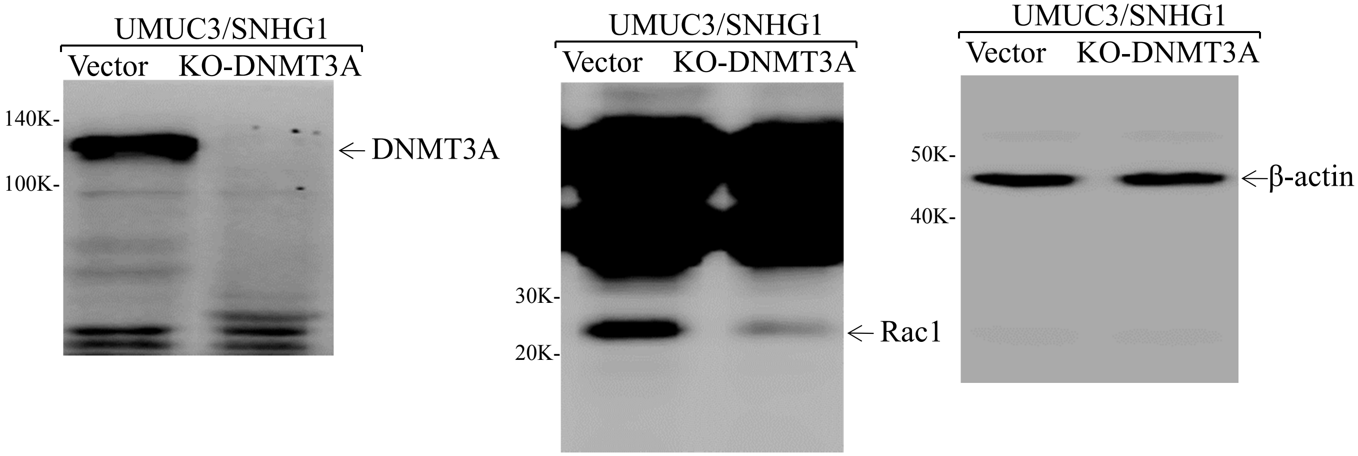

UMUC3/SNHG1
Vector
KO-DNMT3A
140K-
100K-
← DNMT3A
UMUC3/SNHG1
Vector
KO-DNMT3A
30K-
20K-
← Rac1
UMUC3/SNHG1
Vector
KO-DNMT3A
50K-
40K-
←β-actin

Figure6C

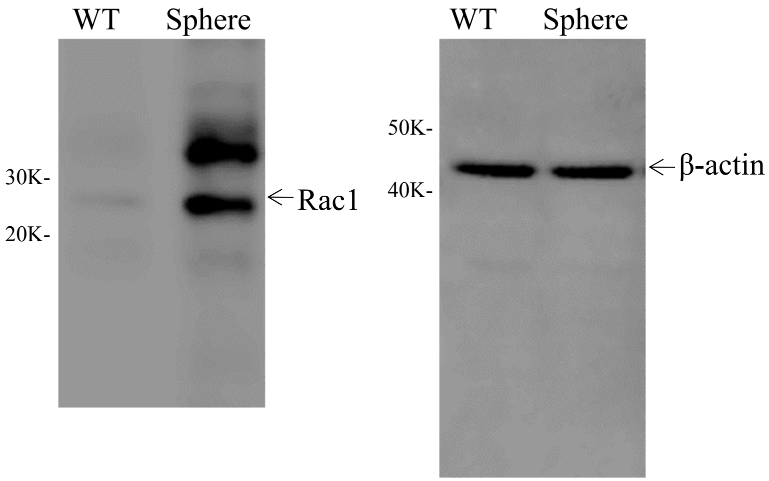

FigureS4C

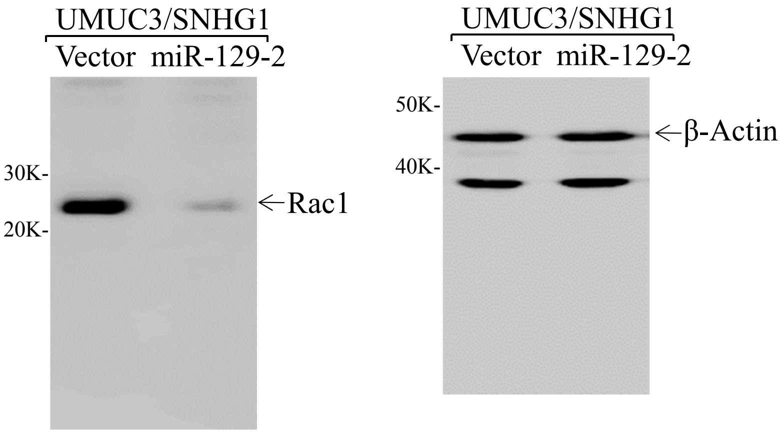
UMUC3/SNHG1
Vector miR-129-2
30K-
20K-
←Rac1
UMUC3/SNHG1
Vector miR-129-2
50K-
40K-
←β-Actin

FigureS5A

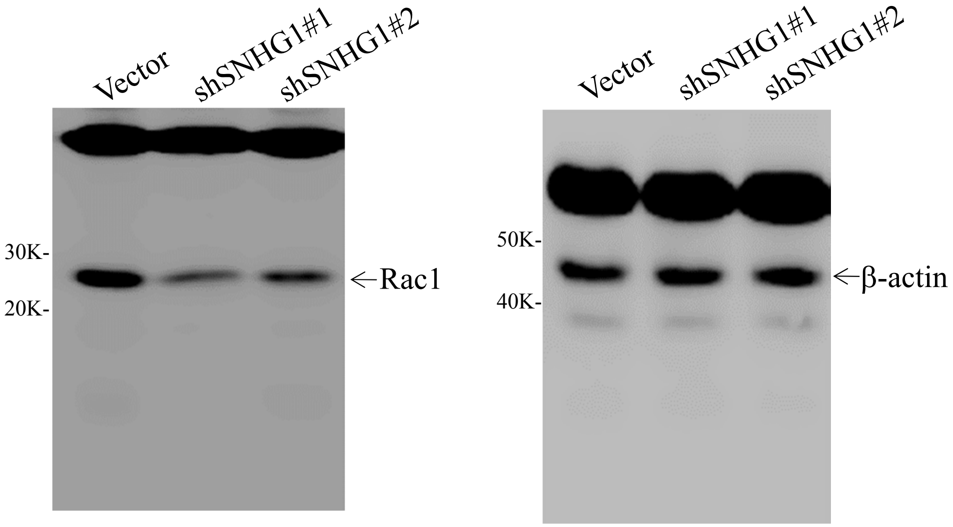
Vector
shSNHG1#1
shSNHG1#2
30K-
20K-
←Rac1
Vector
shSNHG1#1
shSNHG1#2
50K-
40K-
←β-actin
