# Supplementary material for: DNMT3A/miR-129-2-5p/Rac1 Is an Effector Pathway for SNHG1 to Drive Stem-Cell-like and Invasive Behaviors of Advanced Bladder Cancer Cells
Source: Cancers (Basel). 2022 Aug 27;14(17):4159. doi: 10.3390/cancers14174159 (PMC9454896; doi:10.3390/cancers14174159)
Supplement: Supplementary file 1 [file cancers-14-04159-s001.zip › cancers-1867923-supplementary.pdf]

*Supplementary Materials:*

# DNMT3A/*miR-129-2-5p*/Rac1 Is an Effector Pathway for *SNHG1* to Drive Stem Cell-like and Invasive Behaviors of Advanced Bladder Cancer Cells

Jiheng Xu[1,#], Rui Yang[1,#], Jingxia Li[1,#], Lidong Wang[2], Mitchell Cohen[1]
Diane M. Simeone[2,3], Max Costa[1,*] and Xue-Ru Wu[3,4,5,*]

*Send editorial correspondence to:
Dr. Xue-Ru Wu
Department of Urology
New York University School of Medicine
Veterans Affairs Medical Center in Manhattan
423 E23 Street, 6th Floor, Room 6028 West
New York, New York 10010
Tel: 212-263-4167
Fax: 212-951-5424
Email: xue-ru.wu@med.nyu.edu

Or

Dr. Max Costa
Department of Environmental Medicine
New York University School of Medicine
341 East 25th Street
New York, NY, 10010
Tel: 646-469-5298
E-mail: max.costa@nyulangone.org

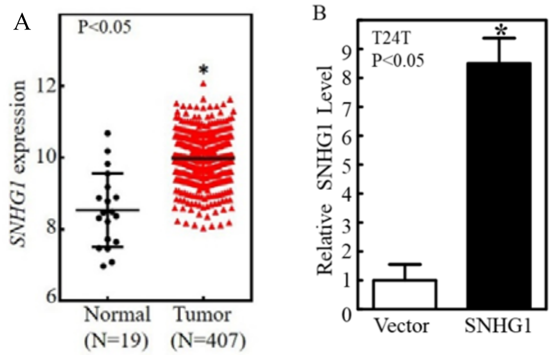


**Figure S1. Expression of *SNHG1* in human bladder cancer and enforced expression of *SNHG1* in bladder cancer cell line.** (**A**) Comparison of *SNHG1* expression in human bladder tumor vs. normal bladder tissues by RNA-seq (Illumina-Hiseq) based on TCGA database. Long and short horizontal lines denote mean and SD, respectively. (**B**) *SNHG1* overexpression was verified in T24T(*SNHG1*) in comparison to T24T(Vector) cells. The asterisk (*) indicates a statistically significant increase of *SNHG1* in T24T(*SNHG1*) cells than in T24T(Vector) cells ($P < 0.05$).

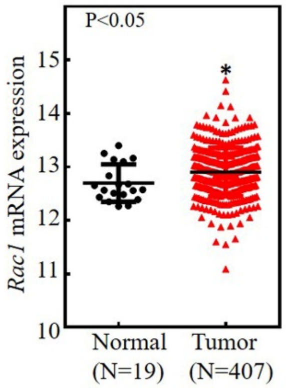


**Figure S2. Expression of *Rac1* mRNA in human bladder cancer.** *Rac1* mRNA expression in human bladder tumor vs. normal bladder tissues by RNA-seq (Illumina-Hiseq) based on TCGA database.

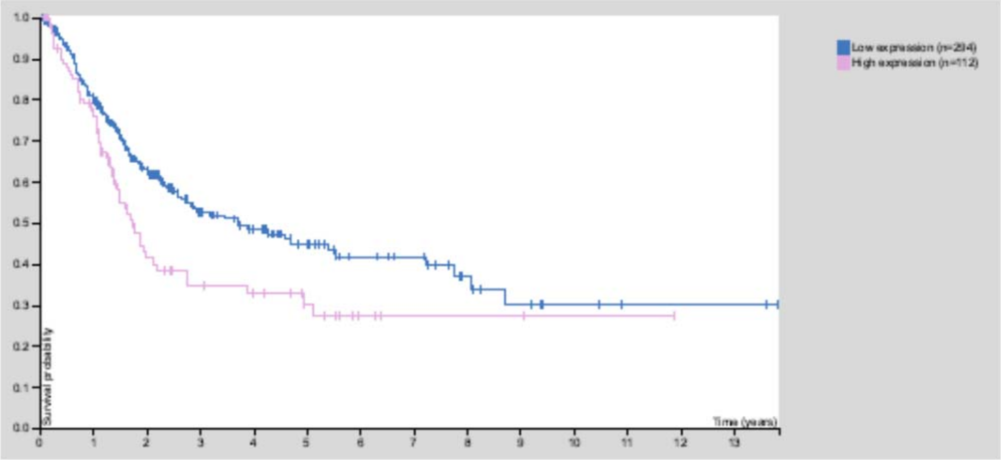


**Figure S3. Rac1 mRNA expression in human bladder cancer.** Analysis of Human Protein Atlas shows that the high expression of Rac1 in human MIBC is strongly associated with poor survival. N=406. P=0.0087.

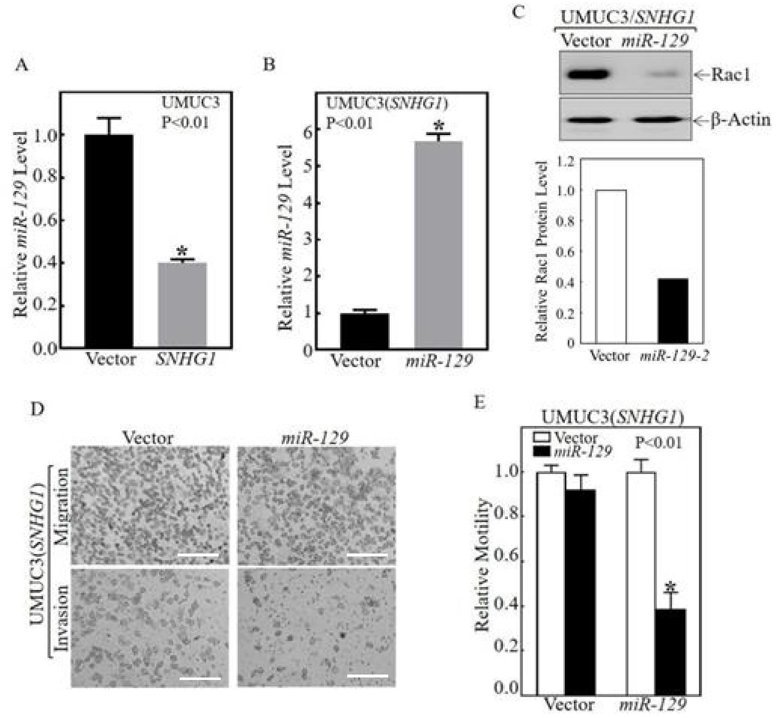


**Figure S4. *SNHG1* overexpression inhibited *miR-129-5p* expression in UMUC3 cells. (A)** Quantitative real-time PCR was carried out to determine the expression levels of *miR-129-2* in UMUC3 cells. **(B)** Plasmids bearing *miR-129-2* were stably transfected into UMUC3(*SNHG1*) cells. The stable transfectants were identified by real-time PCR. (**C, Top panel**) Protein lysates extracted from the indicated cells were subjected to Western blotting to determine Rac1 expression. β-Actin was used as a loading control. **(C, Bottom panel)** Relative protein levels determined by densitometry and expressed as ratios versus β-Actin. (**D-E**) UMUC3(*SNHG1*) cells and UMUC3(*SNHG1*/*miR-129-2*) cells were subjected to a transwell invasion assay, and the migration and invasion were calculated and presented relative to UMUC3(*SNHG1*) control. The results were presented as mean ± SD from triplicate experiments and asterisk (*) indicated a significant decrease in comparison to UMUC3(*SNHG1*) cells ($p < 0.01$). Bars in (D) equal to 100 μm.

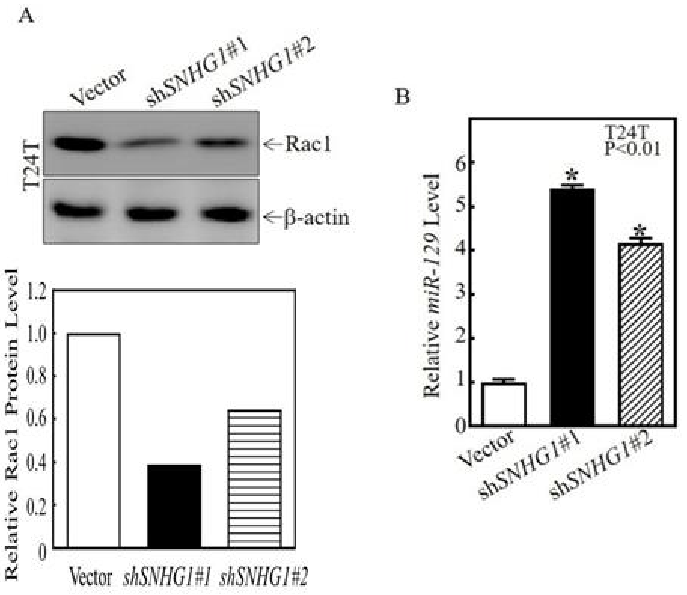


**Figure S5. *SNHG1* inhibition decreased Rac1 expression and promoted *miR-129-5p* expression in UMUC3 cells. (A, Top panel)** The total protein extracts from the indicated cells were subjected to Western blotting to determine for the expression of Rac1 expression. β-Actin served as a protein loading control. (**A, Bottom panel**) Relative protein levels determined by densitometry and expressed as ratios versus β-Actin. **(B)** Quantitative real-time PCR was carried out to determine the expression levels of *miR-129-5p* in the indicated cells. The results were presented as mean ± SD from triplicate experiments and asterisk (*) indicated a significant increase in comparison to T24T(Vector) cells ($p < 0.01$).

**Table S1.** The potential *miRNA* binding sites in *Rac1* mRNA 3'UTR region

| | |
|---|---|
| Position 56-62 of rac1 3' UTR | 5' ...UUUGUACGCUUUGCUCAAAAAAA... |
| hsa-miR-129-5p | 3' CGUUCGGGUCUGGCGUUUUUC ... |
| Position 66-72 of rac1 3' UTR | 5' ...UUGCUCAAAAAAAAACAAAAAAA... |
| hsa-miR-129-5p | 3' CGUUCGGGUCUGGCGUUUUUC |
| Position 79-85 of rac1 3' UTR | 5' ...AACAAAAAAAAAAAACAAAAAAA... |
| hsa-miR-129-5p | 3' CGUUCGGGUCUGGCGUUUUUC |
| Position 987-993 of rac1 3' UTR | 5' ...AACCCCUUCUGACUGAGCAAUAU... |
| hsa-miR-137 | 3' GAUGCGCAUAAGAAUUCGUUAUU |
| Position 308-315 of rac1 3' UTR | 5' ...UGUUCAGAUUAAGAGUUGCCAAA... |
| hsa-miR-182 | 3' UCACACUCAAGAUGGUAACGGUUU |
| Position 537-544 of rac1 3' UTR | 5' ...UCCCGACAUAACAUUGUACUGUA... |
| hsa-miR-101 | 3' AAGUCAAUAGUGUCAUGACAU |
| Position 904-910 of rac1 3' UTR | 5' ...AUGCCUCCCCAAAUUGGGCAUUU... |
| hsa-miR-365 | 3' UAUUCCUAAAAAUCCCCGUAAU |
| Position 309-315 of rac1 3' UTR | 5' ...GUUCAGAUUAAGAGUUGCCAAAA... |
| hsa-miR-96 | 3' UCGUUUUUACACGAUCACGGUUU |
| Position 127-133 of rac1 3' UTR | 5' ...UCAAUGCCAACUUUUUGUUACAG... |
| hsa-miR-194 | 3' AGGUGUACCUCAACGACAAUGU |
